# Supplementary material for: Individual differences in executive functions and theory of mind mediate the relation between academic skills from kindergarten to 5th grade
Source: PLoS One. 2025 Jun 2;20(6):e0324547. doi: 10.1371/journal.pone.0324547 (PMC12129330; doi:10.1371/journal.pone.0324547)
Supplement: Table S1 — (DOCX) [file pone.0324547.s001.docx]

| **Table S1.** Descriptions of the tests measuring other literacy and numeracy skills by grade. | | | | | | | |
| --- | --- | --- | --- | --- | --- | --- | --- |
| Skill | Test | | Description | | Time of testing | | Scoring |
|  |  | |  | | K | 5^th^ grade |  |
|  |  | |  | |  |  |  |
| Vocabulary^1^ | Evaluation du Langage Oral (Khomsi, 2001) | | Determine which of four pictures corresponds to a word given by the experimenter. | | X |  | Number of items correctly answered (0–20) |
| Phonological awareness^1^ | Phonology subtest of the Evaluation Des fonctions cognitives et des Apprentissages (EDA) (Billard & Touzin, 2012) | | Repeat pseudo-words said aloud by the experimenter. There were four two-syllable pseudo-words, four three-syllable pseudo-words and two four-syllable pseudo-words to repeat. | | X |  | Number of correctly repeated syllables (0–28) |
| Pragmatics^1^ | Scalar task (Stiller et al., 2015) | | Make pragmatic inferences by constructing contextually derived “ad-hoc” implicatures, using sets of pictures with contrasting features. | | X |  | Proportion of plausible choices (non-errors) that are revealing of one-feature item choices, i.e., [pragmatic/(pragmatic + logical) responses] |
| Counting knowledge^1^ | Counting task (adapted from Lipton & Spelke, 2005) | | Count twice to the highest number known | | X |  | Highest number reached |
| Quantitative knowledge^1^ | Tokens | | Perform 10 quantitative tasks. Children have to recognize the numerosity of a given set, create a set given a specific numerosity, solve simple non-symbolic arithmetic problems, create a set of the same numerosity as another distant set, compare the numerosities of two sets, recognize the numerosity of a set created by the addition of two other sets, recognize the numerosity of a set created by the subtraction of one set from another, recognize number symbols, recognize the position of a number within a sequence, and use ordinal information to identify a position in a sequence | | X |  | Overall score from 0 to 10 |
| Arithmetic fluency^2^ | Woodcock-Johnson III (Math Fluency subtest) (Woodcock, Mather, McGrew, & Wendling, 2001). | | Solve as many arithmetic operations (addition, subtraction, and multiplication) as possible within 3 minutes. | |  | X | Number of correctly solved operations (0-160). |
|  |  | |  | |  |  |  |
| **Notes.** ^1^, Tests administered individually. ^2^, Test administered in a whole-class setting. | | | | | | | |
|  | |  | |  | | | |

**References**

Billard, C., & Touzin, M. (2012). *Evaluation Des fonctions cognitives et des Apprentissages de 4 à 11 ans.*

Khomsi, A. (2001). *ELO : Évaluation du langage oral*. ECPA, Les Éditions du Centre de psychologie appliquée.

Lipton, J. S., & Spelke, E. S. (2005). Preschool Children’s Mapping of Number Words to Nonsymbolic Numerosities. *Child Development*, *76*(5), 978‑988. https://doi.org/10.1111/j.1467-8624.2005.00891.x

Stiller, A. J., Goodman, N. D., & Frank, M. C. (2015). Ad-hoc implicature in preschool children. *Language Learning and Development*, *11*(2), 176‑190. https://doi.org/10.1080/15475441.2014.927328

Woodcock, R. W., Mather, N., McGrew, K. S., & Wendling, B. J. (2001). *Woodcock-Johnson III tests of cognitive abilities.* Itasca, IL: Riverside.
