# Supplementary material for: Individual differences in executive functions and theory of mind mediate the relation between academic skills from kindergarten to 5th grade
Source: PLoS One. 2025 Jun 2;20(6):e0324547. doi: 10.1371/journal.pone.0324547 (PMC12129330; doi:10.1371/journal.pone.0324547)
Supplement: Table S2 — (DOCX) [file pone.0324547.s002.docx]

| **Table S2.** Descriptive statistics (raw scores). | | | | | | | | |
| --- | --- | --- | --- | --- | --- | --- | --- | --- |
| Skill | Test | Time of testing | | N | Mean | SD | Min | Max |
|  |  | K | 5^th^ grade |  |  |  |  |  |
|  |  |  |  |  |  |  |  |  |
| Reading | EDA | X |  | 94 | 19.72 | 15.87 | 0 | 65 |
|  | EVALEO |  | X | 95 | 222.95 | 58.17 | 94.50 | 395.50 |
| Math | Applied Problems | X |  | 94 | 16.79 | 3.43 | 9 | 29 |
|  | Applied Problems |  | X | 95 | 35.60 | 3.97 | 22 | 43 |
| Short-term memory | Corsi | X |  | 94 | 4.17 | 1.19 | 0 | 7 |
|  | Corsi |  | X | 95 | 6 | 0.83 | 3 | 8 |
| Working memory | Corsi | X |  | 93 | 3.12 | 1.06 | 0 | 6 |
|  | Corsi |  | X | 95 | 5.28 | 0.72 | 3 | 6 |
| Self-regulation | HTKS | X |  | 94 | 38.91 | 11.07 | 3 | 52 |
| Planning | EDA | X |  | 56 | 14.09 | 8 | 0 | 29 |
| ToM | Wellman & Liu | X |  | 93 | 3.48 | 1.32 | 0 | 5 |
|  | SET |  | X | 89 | 9.22 | 2.37 | 3 | 12 |
|  | RME |  | X | 89 | 18.19 | 3.04 | 10 | 26 |
| Sharing | Dictator | X |  | 95 | 2.28 | 2.18 | 0 | 10 |
|  |  |  | X | 95 | 3.19 | 1.84 | 0 | 10 |
| Distributive justice | Resource allocation | X |  | 95 | 8.16 | 2.28 | 3 | 12 |
| Social problem-solving (justice) | SPST | X |  | 95 | 0.71 | 0.97 | 0 | 3 |
| Social problem- solving (flexibility) |  | X |  | 95 | 2.15 | 0.96 | 0 | 3 |
| **Notes.** N vary because some children were absent on the day of the tests. | | | | | | | | |
