# Supplementary material for: Individual differences in executive functions and theory of mind mediate the relation between academic skills from kindergarten to 5th grade
Source: PLoS One. 2025 Jun 2;20(6):e0324547. doi: 10.1371/journal.pone.0324547 (PMC12129330; doi:10.1371/journal.pone.0324547)
Supplement: Table S3 — (DOCX) [file pone.0324547.s003.docx]

| **Table S3.** Sensitivity analyses using separate time point measures for working and short-term memory. | | | | | | |
| --- | --- | --- | --- | --- | --- | --- |
| Predictor | Mediator | Dependent variable | Indirect effect | 95%CI | Indirect/total effect | |
|  |  |  |  |  | |  |
| Math (K) | Working memory (K) | Math (5^th^) | 0.17 | [0.06, 0.33] | | 0.32 |
| Math (K) | Working memory (5^th^) | Math (5^th^) | 0.04 | [-0.02, 0.14] | | 0.07 |
| Math (K) | Short-term memory (K) | Math (5^th^) | 0.013 | [-0.05, 0.06] | | 0.02 |
| Math (K) | Short-term memory (5^th^) | Math (5^th^) | 0.01 | [-0.02, 0.05] | | 0.02 |
