# Supplementary material for: Individual differences in executive functions and theory of mind mediate the relation between academic skills from kindergarten to 5th grade
Source: PLoS One. 2025 Jun 2;20(6):e0324547. doi: 10.1371/journal.pone.0324547 (PMC12129330; doi:10.1371/journal.pone.0324547)
Supplement: Table S4 — (DOCX) [file pone.0324547.s004.docx]

| **Table S4.** Partial correlations between ToM scores measured at both kindergarten and 5th grade and academic outcomes. | | | | | | | | | | | | | | | |  | |
| --- | --- | --- | --- | --- | --- | --- | --- | --- | --- | --- | --- | --- | --- | --- | --- | --- | --- |
|  | | Math (K) | | | Math  (5th) | | Reading  (K) | | | Reading (5th) | | | ToM  (K) | | | ToM  (5th) | |
| Math (K) |  |  | — |  |  |  | |  |  | |  |  | |  |  | |  |
| Math (5th) |  |  | **0.30** |  | — |  | |  |  | |  |  | |  |  | |  |
| Reading (K) |  |  | **0.38** |  | **0.43** |  | | — |  | |  |  | |  |  | |  |
| Reading (5th) |  |  | 0.26 |  | 0.05 |  | | **0.29** |  | | — |  | |  |  | |  |
| ToM (K) |  |  | -0.10 |  | -0.07 |  | | -0.19 |  | | 0.26 |  | | — |  | |  |
| ToM (5th) |  |  | 0.22 |  | 0.14 |  | | 0.14 |  | | 0.20 |  | | 0.26 |  | | — |

**Notes.**Controlling for all executive functions (working memory, planning and self-regulation). Significant correlations (p < .05) are in bold.
