# Supplementary material for: Individual differences in executive functions and theory of mind mediate the relation between academic skills from kindergarten to 5th grade
Source: PLoS One. 2025 Jun 2;20(6):e0324547. doi: 10.1371/journal.pone.0324547 (PMC12129330; doi:10.1371/journal.pone.0324547)
Supplement: Attachment — Submitted filename: Response to Reviewers.pdf [file pone.0324547.s006.pdf]

Response to reviews

We wish to thank the reviewers for their comments on our manuscript. Below, we indicate the responses to the specific issues raised and the corresponding changes that we have made in the revised manuscript.

Reviewer #1

This study investigates the predictive value of early academic skills on later academic outcomes in a French sample, exploring whether this association can be partially explained by domain-general mechanisms—specifically, executive functions (EF) and ToM. The authors find that early math and reading abilities predict later academic success both within and across domains, aligning with previous findings primarily from U.S. samples. They also show that self-regulation, working memory, and ToM mediate these relations.

The study addresses a relevant gap by examining these associations in a French context with more homogeneous early education; however, several points warrant clarification or further discussion:

Response: We thank the reviewer for this positive assessment of our paper.

(1) The authors should discuss ToM development more explicitly. Notably, the period between kindergarten and fifth grade encompasses critical advancements in children's understanding of mental states. Differentiating between basic (first-order) ToM skills and more advanced (higher-order) ToM understanding would help contextualize the chosen measures and interpret the stability (or lack thereof) of ToM abilities.

Response: We have followed the reviewer's suggestion and have now significantly expanded the relevant paragraph in the discussion to describe the development of ToM skills. On p. 28, we now state:

"Interestingly, we assessed ToM both in kindergarten and in 5th grade, yet only the 5th-grade measure mediated the relation between all within- and cross-domains relations. ToM measured in kindergarten was neither associated with early math and reading skills nor with later math skills. This pattern likely reflects the developmental trajectory of ToM abilities. Indeed, children's ToM capabilities develop significantly during the preschool years [157], but continue to evolve throughout elementary school [158,159]. By age 4, most children have acquired basic first-order ToM skills, such as understanding that someone can hold a false belief about the world [157] or attributing basic intentions to others [160]. Even very young children can discriminate basic emotions on faces [161]. However, during development, children progressively acquire more advanced ToM capacities, including second-order and higher-order false beliefs [159], increasingly sophisticated mental state and emotion recognition [127], and interpretation of complex social situations [162]. Previous studies have found relatively weak associations between early basic ToM skills and later advanced ToM abilities [79,147,163], as well as modest correlations between different advanced ToM tasks [101,164]. This developmental progression may explain the lack of correlation between our kindergarten and

5th-grade ToM measures, suggesting that they assess qualitatively different constructs at different developmental stages. Specifically, the Wellman & Liu task used in kindergarten measures relatively 'basic' ToM skills, requiring children to compare their own mental states with those of others—a self-other distinction that is critical in early childhood [165]. This widely-used measure was selected as it appropriately captures ToM capabilities at the preschool stage. By 5th grade, most children may have mastered these first-order skills, necessitating more sophisticated measures. Our 5th-grade assessments—the Reading the Mind in the Eyes test [127,128] and the Story-based Empathy Task [126]—evaluate advanced ToM abilities, including attribution of complex mental states and emotions through the interpretation of social situations. These are likely more directly relevant to communication, collaboration, and emotional regulation in the classroom [76], potentially explaining their stronger mediating role."

(2) On page 6, the authors suggest that the social nature of schools is often overlooked in studies of academic achievement. However, there is a substantial body of research linking ToM to various academic domains (e.g., reading, math, scientific reasoning) and investigating these associations longitudinally (e.g., Lecce et al., 2021; Kloo et al., 2022; Osterhaus et al., 2024; see also Tompkins et al., 2024, for a meta-analysis). This literature should be included in the literature review.

Response: We agree that this statement was unwarranted and have now removed it from the manuscript. On p. 6, we have now included all of the studies pointed out by the reviewer. This revised paragraph reads:

"Critically, schools are also social environments. As such, learning at school is fundamentally a social process [73–77] that involves managing relationships with teachers and peers. Therefore, there may be a relation between academic skills and children's abilities to navigate the social world. These abilities may encompass social cognition skills, which center on the ability to understand our own and others' mental states (e.g., thoughts, intentions, emotions, desires, or beliefs) but also include the ability to perceive social relationships (alliance, friendship, dominance) and form beliefs associated with social categories or gender (e.g., stereotypes). These abilities may also include social behaviors, which describe the behaviors that are needed to develop socially appropriate responses and build positive relationships with others (e.g., sharing, communicating, cooperating, and knowing how to solve social problems). The idea that academic achievement relates to social cognition and behavior is supported by a number of cross-sectional and short-term longitudinal studies. For example, proficiency in Theory of Mind (ToM)—the ability to infer mental states—is linked to greater academic skills (see [78] for a meta-analysis) both in preschool [38,79,80] and in elementary school [81–85]. Other studies have found a relation between children's socio-emotional skills (e.g., emotion recognition and emotion understanding) and math and reading skills in preschool [86–89] and in elementary school [90–92]. Social skills (e.g., cooperating, communicating, and sharing with others, being empathetic, taking responsibility, accepting others) have also been found to be associated with concurrent levels of academic achievement [93–96]"

(3) Additional information about the ToM assessments, particularly at fifth grade, would be helpful. The correlation between the two ToM tasks (r = .30) is modest, and the absence of a

longitudinal correlation between ToM measures from kindergarten to fifth grade is unexpected. This might reflect conceptual differences between the measures (e.g., RMET and SET). I wonder if the decision to consider a single ToM score is really justified. Reporting separate correlations between academic achievement and each ToM task would clarify how each measure relates to academic outcomes.

<u>Response:</u> We thank the reviewer for this comment. We have added in the manuscript an analysis of correlations between academic outcomes and SET and RME taken separately to provide a more detailed picture of associations. On p. 15, we have added a sentence indicating that we will present correlations considering both the composite measure of ToM and each individual measure. This sentence reads:

"However, we also report correlations between each measure taken separately and academic outcomes to assess the specificity of measures of empathy and emotion recognition."

On p. 22, we now report these correlations. Overall, we find significant or near-significant associations between academic outcomes and measures of SET and RME taken separately (with the exception of RME and reading score in kindergarten), which in our opinion still supports the use of a more comprehensive composite score. This edited paragraph now reads:

"Finally, we examined whether ToM and social behavior mediated the relation between early and later reading and math skills. We did not find that social behavior was associated with either math or reading skills in both kindergarten and 5th grade. However, ToM skills measured in kindergarten were predictive of reading skills in 5th grade. The composite score of ToM skills measured in 5th grade was also positively correlated with math and reading skills at both time points. The sizes of these correlations were small to medium (see Table 4). Taken separately, both SET and RME scores were significantly correlated with math scores in 5th grade (SET: r=.32, p=.002; RME: r=.27, p=.01) and reading scores in 5th grade (SET: r=.25, p=.02; RME: r=.35, p<.001). SET scores were also significantly correlated with math scores in kindergarten (r=.35, p<.001) and tended to be associated with reading scores in kindergarten (r=.18, p=.098). RME scores tended to be correlated with math scores in kindergarten (r=.20, p=.056), though no association was observed with reading scores in kindergarten (r=.16, p=.125). Therefore, overall, both measures of empathy and emotion recognition were positively associated with academic outcomes in kindergarten and first grade, supporting the use of a composite score of ToM in 5th grade. Interestingly, there was no correlation between ToM skills measured in kindergarten and 5th grade, suggesting that these tests may measure different constructs (see Discussion)."

(4) As ToM is related to EF, it would be informative to report partial correlations between ToM and academic achievement controlling for EF.

<u>Response:</u> We have now added in the manuscript a supplementary table (Table S4) that includes these partial correlations. Note that, although the correlations between ToM skills in 5th grades and academic outcomes remained positive, they no longer reached significance. While this might suggest overlapping variance between ToM and EF measures in predicting academic outcomes, we interpret these findings cautiously for several reasons.

First, there is substantial theoretical and empirical evidence suggesting that ToM and EF are developmentally intertwined constructs (Blair & Razza, 2007; Devine et al., 2016; Carlson et

al., 2013), with executive functions potentially supporting the emergence and expression of ToM abilities. Therefore, attempting to statistically isolate their unique contributions may create an artificial distinction that does not reflect their integrated development. Second, the statistical control of EF when examining the relation between ToM and academic achievement potentially removes meaningful shared variance that is potentially important for understanding academic learning.

On p. 23, we have added a paragraph about these results. It reads:

"Finally, for exploratory purpose, we report in Table S4 the partial correlations between ToM scores measured in both kindergarten and 5th grade and academic outcomes, controlling for all executive functions (working memory, planning and self-regulation). Although the associations between ToM in 5th grade and academic achievement remained positive, they were no longer significant. This might suggest overlapping variance between ToM and executive function measures in predicting academic outcomes. However, these findings need to be interpreted with caution for two reasons. First, there is theoretical and empirical evidence suggesting that ToM and executive functions are developmentally intertwined constructs [38,101,136], with executive functions potentially supporting the emergence and expression of ToM abilities. Therefore, attempting to statistically isolate their unique contributions may create an artificial distinction that does not reflect their integrated development and may remove meaningful shared variance that is potentially important for understanding academic learning."

<u>Reviewer #2</u>

This manuscript (MS) examines the mediating roles of executive functions (EF) and social cognition and behavior (SCB) in the longitudinal associations between reading and math at kindergarten and 5th grade in 95 children. The MS is generally well-written, in terms of linguistic clarity. The topic is of interest as there are limited studies examining longitudinal pathways of development in diverse settings. However, I have some concerns with the MS in its current state. In particular, conceptual clarity needs to be enhanced in several areas.

1. First, it is unclear and rather perplexing why data from two timepoints several years apart are combined in a composite score. What do the scores represent? How do they fit in a model where, e.g., math scores from the same test at kindergarten and Grade 5 are separate manifest variables? The underlying logic needs to be explicated.

<u>Response:</u> We apologize for the lack of clarity regarding these composite scores. We have revised our manuscript to provide explicit justification for this methodological choice and to clarify the rationale behind our approach.

First, we have added the following explanation in the Methods section. On p. 15, a new paragraph now reads:

“For working memory and sharing behavior, we created composite scores across time points because: (1) identical instruments were used at both time points (Corsi Block Tapping task for working memory measures and Dictator Game for sharing behavior), (2) these measures showed significant correlations between kindergarten and 5th grade assessments ($r = .29$, $p < .005$ for working memory; $r = .34$, $p < .001$ for sharing behavior), and (3) these constructs represent relatively stable individual traits that have demonstrated moderate stability across development in previous longitudinal research [123,124]. Therefore, composite scores represent the general capacity of children in working memory and sharing behavior across the studied developmental period, allowing us to examine how these traits might mediate the relation between early and later academic skills (note that we also created a composite measure of short-term memory so that it was comparable to the composite measure of working-memory). Nonetheless, we also conduct separate mediation analyses using separate time point measures rather than composites to examine the sensitivity of our findings.”

Second, in the Discussion section (p. 31), we have acknowledged the limitations of this approach (while also acknowledging its benefits):

"Second, we decided to use composite scores for certain measures across time points. This carries both advantages and limitations. While this approach allows us to capture more reliable estimates of relatively stable traits (which were significantly correlated across time points), it might obscure potential developmental changes in these capacities. Future research with larger samples might explore more complex models that separate these constructs at different time points while still examining their mediating roles."

Third, we have conducted sensitivity analyses using the separate time point measures instead of the composites to verify the robustness of our findings. These results are now reported in Supplementary Table S3 and summarized in the manuscript (p. 21):

“Because working-memory and short-term memory measures were calculated using composite scores across time points, we also conducted sensitivity analyses using separate time point measures rather than composites (see Table S3). These analyses revealed that kindergarten working memory significantly mediated the relationship between early and later math skills (indirect effect = 0.17, 95% CI [0.06, 0.33], $p = 0.002$), whereas 5th grade working memory showed a smaller and non-significant mediation effect (indirect effect = 0.04, 95% CI [-0.02, 0.14], $p = 0.20$). While the point estimates might suggest potentially stronger mediation effects for working memory measure at kindergarten, the overlapping confidence intervals indicate that we cannot conclusively determine that one time point's measurement provides a significantly stronger mediating effect than the other. Neither kindergarten nor 5th grade short-term memory significantly mediated the relationship between early and later math skills.”

2. The rationale and justification for the mediation models tested should be articulated. Early EF/SCB mediating the link between early to later academic skills is problematic as the early EF/SCB is measured at the same timepoint as the early academic skills. There is much evidence of EF/SCB contributing to academic skills and, as the authors pointed out in their literature reviews, studies have found earlier academic skills predicting later EF/SCB. The current analyses and findings do not rule out, theoretically and empirically, the alternative model where early academic skills mediate the link between early EF/SCB and later academic skills. It is unclear what is being examined in models with the K/5th composite measures. The authors

highlighted literature suggesting bidirectional associations amongst the constructs but did not account for them in their models. The piecemeal approach ignores what might be extensive overlapping variance among the early and late EF, SCB, math, and reading skills.

Response: We appreciate the reviewer's comments regarding the directionality of our mediation models. We agree with the reviewer that our current models cannot rule out alternative causal or bidirectional pathways, and we do not believe making such claim in the manuscript. We would like to clarify that our analytical approach was guided by our pre-registered hypotheses (https://osf.io/8f6tb/?view_only=a0988626c2784a56955a602f2de5563e), which specifically aimed to test whether our data are consistent with a hypothesized model in which executive functions and social cognition mediate the relation between early and later academic skills. We acknowledge that other (non-preregistered) models may be consistent with our dataset, as is often the case in correlational designs. Disentangling between these is challenging and was not the goal of the present study.

Nonetheless, the reviewer raises an important point. We have significantly expanded and strengthened our discussion of the study limitations regarding causality and bidirectionally in the revised manuscript. On p. 31, the revised paragraphs read:

"Third, although the relationship between early and later academic skills is longitudinal, measurements of executive functions, ToM, and social behavior were collected simultaneously with academic skills in kindergarten and 5th grade. Therefore, the relations between our potential mediators and academic skills are often (though not always) cross-sectional. Furthermore, our study is correlational, and our findings do not establish the causal aspects of the mediation models. It is important to emphasize that our mediation analyses were designed to test specific pre-registered hypotheses about whether domain-general factors help explain the relationship between early and later academic skills, not to establish exclusive causal pathways. While the observed relations are consistent with our model, alternative models could also explain our results. For example, it is possible that executive functions and ToM causally influence early academic skills, which in turn may influence later academic skills.
Truly disentangling bidirectional relationships between cognitive functions and academic skills presents significant methodological challenges, particularly with observational data collected at only two time points. While alternative analytical approaches such as cross-lagged panel models could potentially provide additional insights into these bidirectional relationships, such models typically benefit from at least three measurement time points for optimal estimation of developmental trajectories [169,170] and would be statistically challenging given our sample size [171]. In a mediation framework such as the one followed here, the choice of the most plausible mediation model ultimately depends on prior findings, logical reasoning, and theoretical considerations [172]. Our approach follows methodological precedents in this literature (e.g., [68,118]) while acknowledging that the observed relations likely reflect complex, reciprocal developmental processes.
Importantly, our model is grounded in a general theoretical framework that has guided several studies since the seminal meta-analysis by Duncan et al. (2007) [21,68,118]. Nevertheless, intervention studies are needed to substantiate causal claims, and our findings should be viewed as an initial critical test of the models discussed. Future studies with larger samples and additional measurement time points could employ alternative analytical approaches to more comprehensively examine potential bidirectional relationships between executive functions, theory of mind, and academic skills across development."

We believe this clarification preserves the integrity of our pre-registered analytical approach while openly acknowledging its limitations regarding causal inference. Given our sample size limitations, we believe that attempting to fit more complex bidirectional models would significantly reduce statistical power and potentially lead to less reliable conclusions. Instead, we have focused on providing a clear test of our primary hypotheses while transparently discussing alternative interpretations of the observed relationships.

3. It is strongly recommended that the authors reconsider the appropriate analytical model that will address the research gaps/questions in a more comprehensive and conclusive manner, such as a cross-lag model that considers concurrent and longitudinal autoregressive and cross-lag associations amongst EF, SCB, reading and math. There may be power limitations with the small sample size; the authors may want to sharpen their focus. As the authors highlighted, a key strength of the study lies in its multiple timepoints and measures. However, the current analysis of this valuable dataset falls short of realizing its full potential.

Response: We thank the reviewer for suggesting alternative analytical approaches such as cross-lagged panel models. We appreciate the methodological recommendation and agree that such models can provide valuable insights into bidirectional relationships when appropriately applied.

After careful consideration, we have maintained our pre-registered mediation analysis approach for three reasons. First, our study was specifically designed to test the mediating role of executive functions and theory of mind in the relation between early and later academic skills, as explicitly stated in our pre-registered hypotheses (https://osf.io/8f6tb/?view_only=a0988626c2784a56955a602f2de5563e). Deviating from this analytical plan would contradict the principles of pre-registration. Second, cross-lagged panel models, while valuable for examining bidirectional relationships, have different statistical requirements and interpretive frameworks than our mediation approach. Importantly, they typically benefit from at least three measurement time points for optimal estimation of developmental trajectories, whereas our study includes two primary measurement periods. Given our sample size (n=95), fitting cross-lagged models with multiple variables would significantly reduce statistical power and potentially lead to estimation issues, particularly for complex constructs with multiple indicators. Third, the developmental timing of our measurements (kindergarten and 5th grade) represents a substantial gap that may not be optimally suited for cross-lagged analyses, which typically benefit from more closely spaced measurement occasions.

Nevertheless, we acknowledge the value of the reviewer's suggestion for future research. We now discuss cross-lagged panel models in our limitations section (p. 31):

“Third, although the relationship between early and later academic skills is longitudinal, measurements of executive functions, ToM, and social behavior were collected simultaneously with academic skills in kindergarten and 5th grade. Therefore, the relations between our potential mediators and academic skills are often (though not always) cross-sectional. Furthermore, our study is correlational, and our findings do not establish the causal aspects of the mediation models. It is important to emphasize that our mediation analyses were designed to test specific pre-registered hypotheses about whether domain-general factors help explain the relationship between early and later academic skills, not to establish exclusive causal

pathways. While the observed relations are consistent with our model, alternative models could also explain our results. For example, it is possible that executive functions and ToM causally influence early academic skills, which in turn may influence later academic skills.
Truly disentangling bidirectional relationships between cognitive functions and academic skills presents significant methodological challenges, particularly with observational data collected at only two time points. While alternative analytical approaches such as cross-lagged panel models could potentially provide additional insights into these bidirectional relationships, such models typically benefit from at least three measurement time points for optimal estimation of developmental trajectories [169,170] and would be statistically challenging given our sample size [171]. In a mediation framework such as the one followed here, the choice of the most plausible mediation model ultimately depends on prior findings, logical reasoning, and theoretical considerations [172]. Our approach follows methodological precedents in this literature (e.g., [68,118]) while acknowledging that the observed relations likely reflect complex, reciprocal developmental processes.
Importantly, our model is grounded in a general theoretical framework that has guided several studies since the seminal meta-analysis by Duncan et al. (2007) [21,68,118]. Nevertheless, intervention studies are needed to substantiate causal claims, and our findings should be viewed as an initial critical test of the models discussed. Future studies with larger samples and additional measurement time points could employ alternative analytical approaches to more comprehensively examine potential bidirectional relationships between executive functions, theory of mind, and academic skills across development."

We believe this approach respects the value of the reviewer's methodological suggestion while maintaining the scientific integrity of our pre-registered analytical framework.

Reviewer #3

This paper reports on a longitudinal study on the development of reading and math from kindergarten to fifth grade, investigating cross-domain associations and mediation affects of domain-general cognitive skills executive functions and theory of mind. It's commendable that the authors pre-registered their analyses and have shared their data. The paper is clearly written and the results and conclusions are straightforward and consistent with prior research. I have only two minor comments.

Response: We thank the reviewer for this positive assessment of our manuscript.

First, the figure resolution is very poor in this submitted version and should be addressed.

Response: We have now uploaded higher resolution figures in the revised version of the manuscript.

Second, the hypothesis of universal pre-k potentially reducing variability in kindergarten math performance is a very interesting and important question, but not sufficiently engaged with in the current paper. The authors rightfully note in the discussion section that the pattern of results they found could either be explained by inequalities in access to high quality early education (or in other words failure of policy implementation), or cognitive factors or other difference

that cannot be changed by school (what could those be? genetic influences?). Research on the debates of the long run impacts of early education are discussed in the paper (e.g. Burchinal et al., 2024), but it's not clear how the current paper contributes to this debate given the findings are inconclusive. What would be needed to tease apart these competing explanations? What are the future research directions? While I acknowledge the need to replicate findings in more diverse school systems and culture, I am not convinced these findings really have anything to offer to this particular debate and the way it is currently laid out is very speculative. The authors may be better off focusing on the more novel finding that ToM emerged as a significant predictor.

Response: We agree that this aspect of our result and of our discussion was more speculative than the data warranted. In our revised manuscript, we have substantially modified our discussion of this issue, following the reviewer's feedback. Notably, we have removed the entire section that was entitled "A relation between early and later academic skills despite homogenous preschool education" to avoid overinterpretation of our cross-cultural comparison. Instead, we have revised the opening paragraph of our discussion to present a more balanced interpretation of our findings while redirected emphasis to our empirical contribution regarding domain-general mediating factors.

On p. 24, this revised paragraph reads:

"The present study aimed to both replicate the relation between early and later academic skills in a sample of French children followed longitudinally from kindergarten to 5th grade, but also to shed some light on the mechanisms that may underlie that relation. Our findings largely replicate the prior evidence that there are within- and cross-domain relations between early and later reading and math skills, with associations of comparable magnitude to those observed in studies from the United States (e.g., [6,7]). Therefore, although we initially hypothesized that the more uniform early education system in France might attenuate these relations, our data do not support this conclusion (see consistent data in Norway [68] and Finland [137]). This might be because French preschool education struggles to equalize opportunities and might not provide high quality early education to all children [138], which may exacerbate individual differences in skill-building. Alternatively, it could indicate that the relation between early and later academic skills is driven by environmental factors (e.g., home learning environments; [139] and genetic factors [140]) that are relatively independent from early education. Nonetheless, our study provides empirical evidence that domain-general factors such as self-regulation, working memory, and ToM partially mediate the relation between early and later academic achievement, suggesting that multiple developmental pathways likely contribute to academic continuity over time."
